# “Give, but Give until It Hurts”: The Modulatory Role of Trait Emotional Intelligence on the Motivation to Help

**DOI:** 10.1371/journal.pone.0130704

**Published:** 2015-06-29

**Authors:** Sergio Agnoli, Andrea Pittarello, Dorina Hysenbelli, Enrico Rubaltelli

**Affiliations:** 1 Marconi Institute for Creativity, MIC, University of Bologna, Bologna, Italy; 2 Department of Developmental and Socialization Psychology, University of Padova, Padova, Italy; 3 Center for Cognitive Neuroscience, University of Padova, Padova, Italy; University of Tuebingen Medical School, GERMANY

## Abstract

Two studies investigated the effect of trait Emotional Intelligence (trait EI) on people’s motivation to help. In Study 1, we developed a new computer-based paradigm that tested participants’ motivation to help by measuring their performance on a task in which they could gain a hypothetical amount of money to help children in need. Crucially, we manipulated participants’ perceived efficacy by informing them that they had been either able to save the children (positive feedback) or unable to save the children (negative feedback). We measured trait EI using the Trait Emotional Intelligence Questionnaire–Short Form (TEIQue-SF) and assessed participants’ affective reactions during the experiment using the PANAS-X. Results showed that high and low trait EI participants performed differently after the presentation of feedback on their ineffectiveness in helping others in need. Both groups showed increasing negative affective states during the experiment when the feedback was negative; however, high trait EI participants better managed their affective reactions, modulating the impact of their emotions on performance and maintaining a high level of motivation to help. In Study 2, we used a similar computerized task and tested a control situation to explore the effect of trait EI on participants’ behavior when facing failure or success in a scenario unrelated to helping others in need. No effect of feedback emerged on participants’ emotional states in the second study. Taken together our results show that trait EI influences the impact of success and failure on behavior only in affect-rich situation like those in which people are asked to help others in need.

## Introduction

When we face others in need, we are assaulted by a variety of feelings. The decisions and the actions we adopt to help suffering people depend on a number of affect-laden factors. These affective factors result from the characteristics of the people who need help (e.g., race, gender, number) [1–4], the cause of suffering [5], the way the situation is presented [6–7], the individual differences in regulating emotional experiences [8], and previous individual experiences in helping others [9].

A large amount of research has explored the affective mechanisms of helping behavior or, broadly speaking, of prosocial behavior. This research indicated feelings like empathy [10], sympathy, or compassion [1–2] as determinants of helping behavior. A scientific debate about the nature of these feelings is rooted on whether helping others in need depends either on selfish or selfless reasons [10–12]. One line of research suggests that the main determinant of helping behavior is the ability to feel empathy, hypothesizing that an altruistically-motivated observer should be mainly concerned with reducing others’ suffering [10,13–14]. An alternative explanation, however, focuses on the motivating facets of the negative arousal of the self [15]. According to this view, the negative arousal caused by others' suffering prompts people to engage in helping behavior in order to feel better. From this perspective, helping behavior due to a negative emotional state is seen as motivated by self-focused feelings. The reduction of an aversive emotional state is a form of emotion regulation that motivates action in the interest of making oneself feel better [16]. According to Dickert and colleagues [16], donation behavior involves two distinct stages. Specifically, the initial decision to help is primarily determined by mood management mechanisms, whereas the subsequent decision on the amount of help is based on empathy-related feelings.

According to this affect-as-information hypothesis, when we face the possibility of helping others in need, we rely on our affective reactions as a source of information to make evaluations on the most effective behavior. Furthermore, the dual information processing framework [17–21] seems particularly useful in explaining the role of affective and deliberative aspects of the decision to help others in need. According to this theoretical approach, human information processing is characterized by two qualitatively distinct yet interconnected processing modes: an immediate and intuitive process (System 1), and a slower and deliberative process (System 2) that can control the output of the first process [18]. System 1 is hypothesized to be the basis of the affective reactions experienced toward the victim. System 2 can interfere with the output of System 1 through deliberative reasoning about the victims and the situation, insomuch as this interference can reduce the impact of immediate affective reactions. Given that the affective reactions provoked by the victims vary, deliberative mechanisms can vary as well, as recently demonstrated by work investigating the ability to understand and draw meaning from numbers [22–24]. The deliberative aspect involved in reasoning about a helping situation can also emerge in the “drop in the bucket” effect [25–26], where victims’ suffering is seen as part of a bigger problem and people’s ability to help is perceived as low, resulting in a reduction of the willingness to help and a smoothness of affective reactions experienced toward the situation.

## Trait Emotional Intelligence

As stated above, a vast amount of research has been dedicated to explaining the main affective determinants of helping behavior. However, several questions regarding the role of individual differences when people are called upon to deal with their own emotions remain unanswered. Why, under identical conditions, are some individuals more willing and motivated to help than others? The answer to this question cannot simply fall under the unspecific theoretical umbrella of individual differences. Therefore, in the present study we tackle this question by suggesting that trait emotional intelligence can explain some individual differences emerging in helping situations. Emotional intelligence (EI) is a recent construct that has been introduced to study social behavior. EI has been proposed as a new perspective in the study of emotions; in particular, this approach maintains that the intelligent use of emotions is essential to explaining both physical and psychological individual adaptation [27]. In EI literature, a major theoretical distinction exists between trait EI (or trait emotional self-efficacy) and ability EI (or emotion-related cognitive abilities) [28]. The former is measured through self-report questionnaires, whereas the latter is measured through performance-based tests [28]. While personality tests capture typical performance, ability tests aim to capture maximal performance [29].

The major criticism of trait models of emotional intelligence is their redundancy of established personality trait taxonomies [30]. Petrides and Furnham performed a systematization of early works on trait EI and proposed a model that summarizes all personality aspects related to affect into a single framework of trait EI, or trait emotional self-efficacy [31–32]. Trait EI is defined as a set of emotional perceptions located at the lower level of personality hierarchies [28]. The concept of trait EI basically proposes that individuals differ in the way they process, use, and regulate affect-laden information of intrapersonal or interpersonal nature [29]. Essentially, this concept recognizes the subjective nature of human emotional experience and is concerned with people’s perceptions of their own emotional abilities. This model has demonstrated good discriminant validity in relation to personality by being independently located in both Eysenck’s and Five Factor spaces [33]. Moreover, numerous studies demonstrated the incremental validity of trait EI over personality taxonomies in relation to criteria such as: happiness [34–36], wellbeing and job satisfaction [37], somatic complaints and life satisfaction [38], sensitivity to stress induction and mood changes [39], personality disorders, depression, coping, and rumination [28,33,40]. These studies demonstrated the construct’s predictive power for many psychological phenomena over and above personality dimensions and personality-related constructs, and suggested that trait EI theory can be employed as a framework to explain individuals’ variability in relation to affect-related criteria. Behavioral-genetic investigations provided further evidence for the conceptualization of trait EI as an independent personality trait. In a study on mixed dizygotic and monozygotic twins, Vernon, Villani, Schermer, and Petrides found that the whole phenotypic associations between trait EI and the Big Five factors were attributable, primarily, to correlated genetic factors and, secondarily, to correlated non-shared environmental factors [41].

As far as the Petrides’s model is concerned, then, empirical findings have failed to support Matthews and colleagues’ criticism. Recently, the study of trait EI has also revealed new insights into the study of decision-making [42–43]. High trait EI individuals are thought to be more able to manage stress and peer relations [44], and are considered more sensitive to environmental emotional cues. Moreover, high trait EI individuals are more likely to show positive affective states than low EI counterparts, thanks to more effective emotion regulation and management mechanisms [45–46]

## Study 1: Trait EI and helping behavior

In the present study, we explore the role of trait EI on helping behavior by devising a new computer-based paradigm that tested participants’ implicit motivation to help. This new methodology measures participants’ accuracy and reaction times in a task that required them to help children in need by clicking on their pictures. Participants were informed that by clicking accurately on the pictures of some children within 500 milliseconds (ms) of their appearance on the screen, they would be able to earn an amount of money sufficient to save the life of the children. The position of the picture varied across trials; specifically, the picture appeared randomly on one of the four different corners of the screen. Such a paradigm measures motivation without explicitly asking for participants’ willingness to help by inferring it from individuals’ performance (i.e., from their accuracy in clicking on children’s pictures and/or from their response reaction times). Differences in accuracy and reaction times represented a measure of participants' implicit motivation to help, with grater effort indicating a stronger motivation to help. Participants completed five blocks with 30 trials in each block. In order to manipulate participants’ perceived efficacy, we introduced two between-subject conditions by presenting positive or negative feedback after each block, informing participants they had been either able (positive feedback condition) or unable (negative feedback condition) to save the child presented in that block, irrespective of their actual performance. Through this manipulation, we intended to study the role of perceived efficacy, and the related affective reactions, on the motivation to help. We measured affective reactions using the Positive Affect and Negative Affect Schedule–Expanded Form (PANAS-X) [47], which participants completed at the beginning of the experiment and at the end of each block. Additionally, we measured trait EI using the Trait Emotional Intelligence Questionnaire–Short Form (TEIQue-SF) [48].

Our specific hypotheses regarding the role of affect and trait EI on participants’ helping behavior as measured by the task performance were as follows:

H1) The presentation of negative feedback (“you did not save the child”) should induce a sense of inefficacy, prompting negative affective states of increasing intensity across blocks; on the contrary, the presentation of positive feedback (“you saved the child”) should induce an increasing sense of efficacy, intensifying positive affective reactions.

H2) We expected performance to vary across conditions, with a reduced performance in the negative condition and an enhancement or maintenance in the positive one; in particular, an increased sense of inefficacy should induce a reduction in the willingness to help, determining an increase in reaction times and a decrease in accuracy.

H3) Trait EI should modulate participants’ affective reactions;. in particular, while positive feedback should strongly affect low trait EI participants, inducing more intense positive affective reactions across blocks, it should not induce more intense positive affective states in high trait EI participants, due to their usually higher positive affective disposition.

H4) Trait EI should moderate the affective states induced by the feedback: in particular, although the negative feedback should prompt a sense of inefficacy and negative affective reactions in all participants, we hypothesized that people with high trait EI could manage their impact, allowing a better performance across blocks (a reduction in reaction times and an increase in accuracy) than low trait EI individuals.

### Method

#### Ethical statement

The procedure was approved by the Ethical Committee for Research in Psychology (Area 17) of the University of Padova (protocol 1249 N. 5D60A256530D615E135C5B9017C34611).

#### Participants

Sixty-three undergraduate students (55.6% females; mean age 24.1 years, *SD* = 2.2) participated in the study. They were all volunteers and were recruited during class hours. They were then scheduled for individual experimental sessions in the laboratory. Written consent was obtained from each participant upon their arrival at the laboratory as required by the regulation of the Ethical Committee of the University of Padova regarding cognitive/behavioral studies involving adult human participants. No participant declined to participate and no participant displayed distress or dropped out of the study.

Participants were randomly assigned to one of the two experimental conditions: 32 participants to the positive feedback condition and 31 to the negative feedback condition. The participants were asked to complete the experimental task, answer a set of questionnaires, and provide demographic information about their gender and age.

#### Questionnaires

The TEIQue-SF is a 30-item scale that measures trait emotional intelligence using a seven-point scale ranging from 1 (“completely disagree”) to 7 (“completely agree”) [48]. The items ask to self-report one’s ability in regulating, expressing, and perceiving his/her emotions. Items in the TEIQue-SF include sentences such as the following: “Expressing my emotions with words is not a problem for me” and “On the whole, I’m pleased with my life.” The final score of the TEIQue-SF ranged between 110 and 189 (*M* = 149.46; *SD* = 17.80). The test showed a good reliability: α = .83.

The PANAS-X is a 60-item, expanded version of the PANAS [47,49]. The scale consists of a number of words that describe different feelings. Participants rated the intensity of the feelings they experienced on a 5-point scale ranging from 1 (very slightly) to 5 (extremely). In addition to the two original higher order scales (positive and negative affect), PANAS-X measures 11 specific kinds of affect: fear, sadness, guilt, hostility, shyness, fatigue, surprise, joviality, self-assurance, attentiveness, and serenity. In the present study we used only 30 items, measuring the sadness, guilt, fatigue, joviality, and self-assurance affect. According to this approach [47], the measure of specific affective states allowed for a more fine-grained analysis of the participants' affective reactions than the more general and unspecific positive and negative affect dimensions. For the sadness, guilt, joviality, and self-assurance affect, the reliability of the scale was very high, ranging from .79 to .94. For the fatigue dimension, reliability was slightly lower but acceptable, ranging from .62 to .72 in the different moments it was measured throughout the experiment (see Table 1 for reliability measures of the PANAS-X scales).

**Table 1:** Reliability (Cronbach’s alpha) of the PANAS-X affect scales administered at the beginning of Study 1 (PRE) and at the end of the five blocks.

PANAS-X Affect	PRE	Block 1	Block 2	Block 3	Block 4	Block 5
Sadness	.883	.905	.878	.878	.867	.850
Guilt	.794	.918	.898	.924	.925	.932
Joviality	.870	.918	.863	.922	.938	.926
Self Assurance	.834	.843	.897	.907	.813	.916
Fatigue	.643	.631	.616	.638	.719	.632

#### Procedure

The experimental session took about 30 minutes and included both a computer task and paper and pencil tasks. Upon arrival at the laboratory, participants were asked to answer the 30 items from the PANAS-X scale [47]. In this first phase of the experiment, individuals were instructed to rate the intensity of their feelings according to "how they felt at that present moment" [49]. Subsequently, they were told that they would participate in a computer task. E-Prime 1.1 software was adopted for the experimental session. Stimulus presentation was conducted with a desktop computer with a 2.4 GHz processor and a 21-inch monitor. A refresh rate of 60 Hz and a resolution of 1440 x 900 pixels were used. Participants were informed that they would be presented with five different experimental blocks, plus a shorter practice block in order to become familiar with the procedure. In each block, a black fixation cross was displayed in the center of the screen for 500 ms followed by a picture of a child (154 x 168 pixels), randomly displayed for 1000 ms either in the top left, top right, bottom right, or bottom left corner of the computer screen.

Each block included 30 trials. The picture of the child did not vary within the same block, but was different across the five experimental blocks. Participants were instructed to click with the left button of the mouse on the child’s picture. In addition, they were told that, in each block, they would earn a hypothetical amount of money to help the child displayed on the screen if the response time of accurate trials was less than or equal to 500 ms (see **S1 File)**.

Two experimental conditions were manipulated between participants as following. In the negative feedback condition (N = 31), participants constantly received the same feedback at the end of each of the five blocks informing that they did not succeed in accurately clicking on the child’s pictures within the time limit, thus being unable to help the child (“You did not save the child”). In the positive feedback condition (N = 32), the same feedback at the end of each block constantly stated that they succeeded in accurately clicking on the pictures within the time limit, thus being able to help the child (“You saved the child”). In both conditions, feedback presentation at the end of each block was preceded by a slide on screen for 3000 ms informing participants that their results were being computed.

Note that although participants’ performance was real, the feedback provided was bogus; it did not reflect participants' actual performance and was adopted solely to manipulate the different experimental conditions. At the end of each block, individuals in both conditions were asked to complete the 30 items of the PANAS-X scale, rating the intensity of the feelings they were experiencing at the present moment [49]. The role of the emotional status monitoring during the task was twofold: to collect data on participants’ feelings at the end of each block; to monitor participants’ affective state, in order to suspend the experiment in the case of an emerging distress state. Finally, at the end of the computer task, participants answered the 30 items of the TEIQue-SF. Before leaving the laboratory, participants were debriefed and thanked. The procedure was approved by the Ethical Board for Psychological Research of the University of Padova.

### Results

Since trait EI was measured at the end of the computer task, a first control on the influence of the feedback condition on trait EI levels was performed. No difference in trait EI emerged between participants in the positive feedback condition and participants in the negative one, *t* (61) = .44, *p* = .662, showing that there was no significant influence of the situational condition on the participants’ emotional disposition (i.e., trait EI).

#### Performance

A first set of analyses explored the influence of feedback valence and trait EI on task performance (accuracy and reaction times). Two split-plot ANOVAs were performed on accuracy and reaction time using Repetition (5 levels) as within-subject factor, and Feedback (positive, negative) and trait EI level (median split in high and low level) as between subjects factors. Additional ANOVAs were used to explore interaction effects. Even if TEIQue provides a continuous measure of trait EI level, it is a common practice in the analysis of trait EI to split the TEIQue scores in high and low level (see [29,44]).

In the analysis performed on response accuracy, an effect of Feedback emerged, *F* (1, 59) = 4.11, *p* = .047, η^2^_p_ = .065, showing greater accuracy in the positive feedback condition (*M* = .97, *SD* = .04) than in the negative feedback condition (*M* = .93, *SD* = .09), thus supporting H2. Moreover, a Repetition X Feedback X trait EI level interaction emerged, *F* (4, 236) = 3.34, *p* = .011, η^2^_p_ = .054.

**Fig 1:**
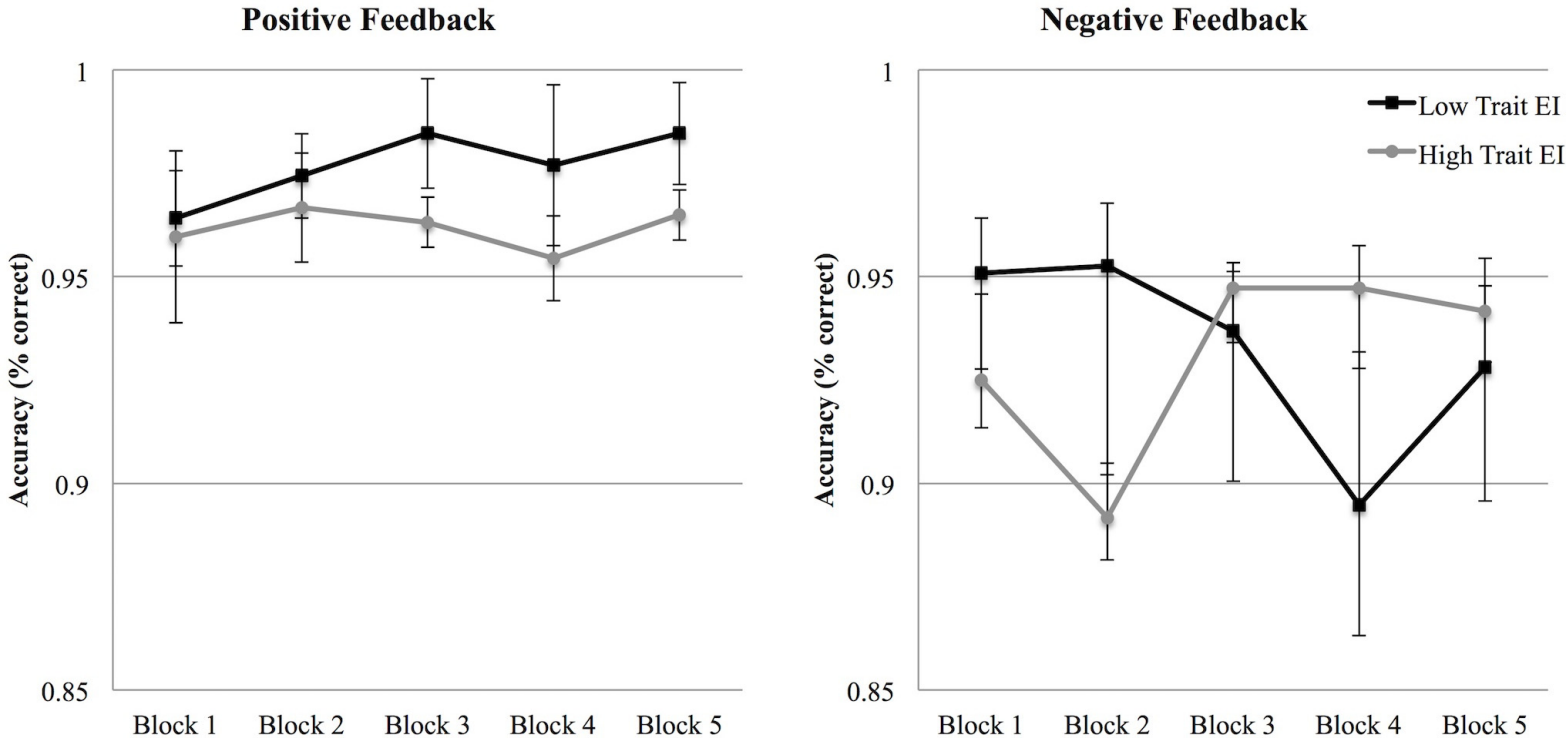
Accuracy (Study 1). Accuracy percentages in the negative (left panel) and in the positive (right panel) feedback conditions across the 5 blocks for low and high trait EI participants. Error bars indicate 1 standard error above or below the mean.

In order to explore this interaction, we performed two separate ANOVAs, one for the positive feedback condition and one for the negative feedback condition, respectively. A Repetition X trait EI level interaction emerged in the negative feedback condition, *F* (4, 116) = 3.24, *p* = .015, η^2^_p_ = .100. As shown in the left panel of Fig 1, the negative feedback produced two different performance trends in low and high trait EI participants. Fully supporting H4, while low trait EI participants receiving negative feedback reduced their response accuracy across blocks, high trait EI participants, after an initial accuracy decrease, increased their performance until, after the third block, they outperformed low trait EI individuals. Simple effect analysis showed that the most important differences emerged in high trait EI individuals between the first and the third block, *t*(11) = 2.53, *p* = .028, d = 1.53, between the second and the third block, *t*(11) = 4.00, *p* = .002, d = 2.41, and between the second and the fourth block, *t*(11) = 3.63, *p* = .004, d = 2.19 (see Fig 1, left panel). No significant effect emerged in the positive feedback condition (Fig 1, right panel).

**Fig 2:**
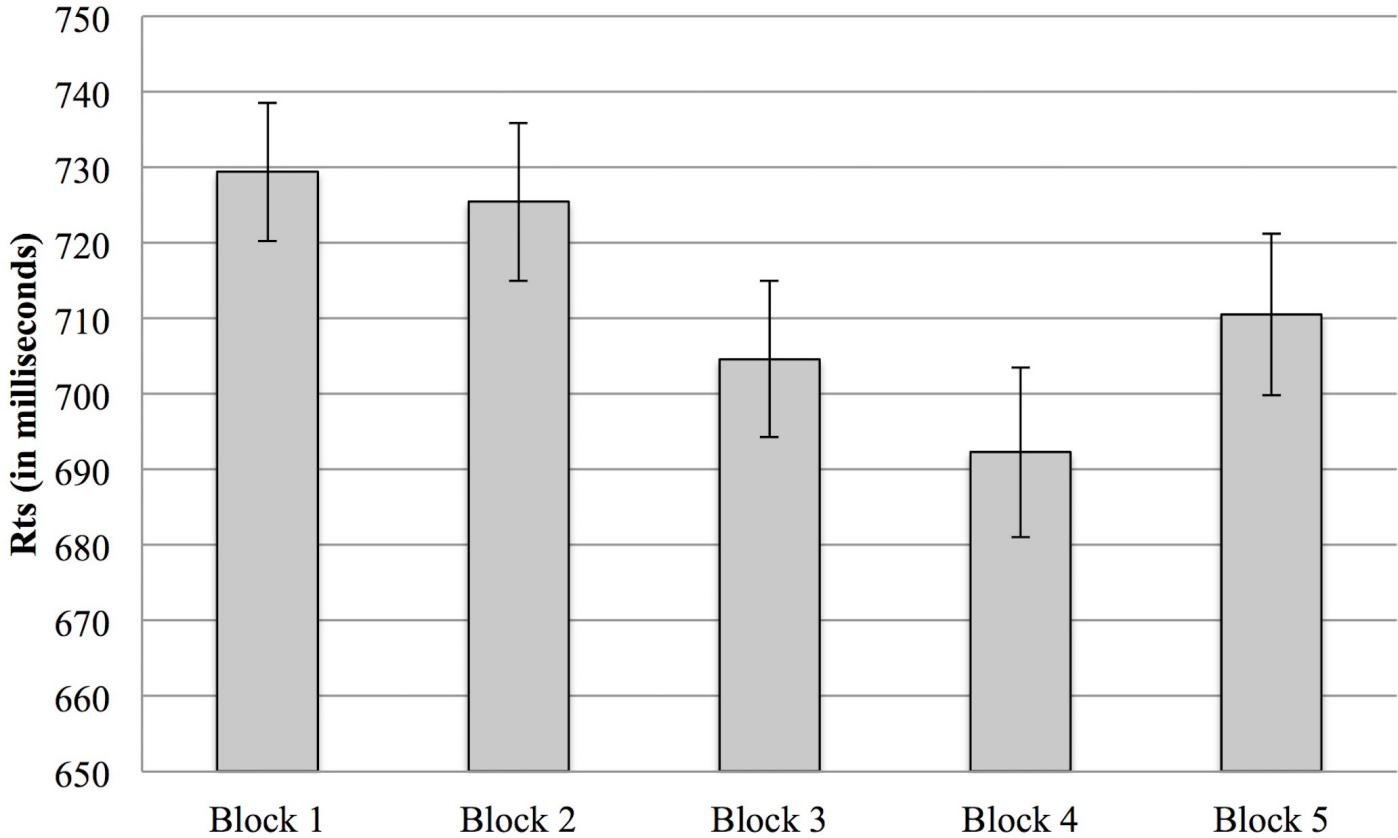
Reaction times (Study 1). Response reaction times in the 5 trials blocks. Note: * α < .01, **α < .001. Error bars indicate 1 standard error above or below the mean.

The analysis performed on reaction times showed only a Repetition effect, *F* (4, 236) = 13.89, *p* < .001, η^2^_p_ = .191, which highlighted a general reduction in reaction times across blocks (Fig 2). No effects of feedback condition or of trait EI emerged.

#### Affect

The same statistical design was adopted also for the analyses of affective dimensions. A series of split-plot ANOVAs were performed on self-rated affective dimensions (sadness, guilt, fatigue, joviality, and self-assurance) using Repetition (6 levels) as within subject factor and Feedback (positive vs. negative) and trait EI level (high vs. low) as between subject factors. Additional ANOVAs were used to explore interaction effects.

**Fig 3:**
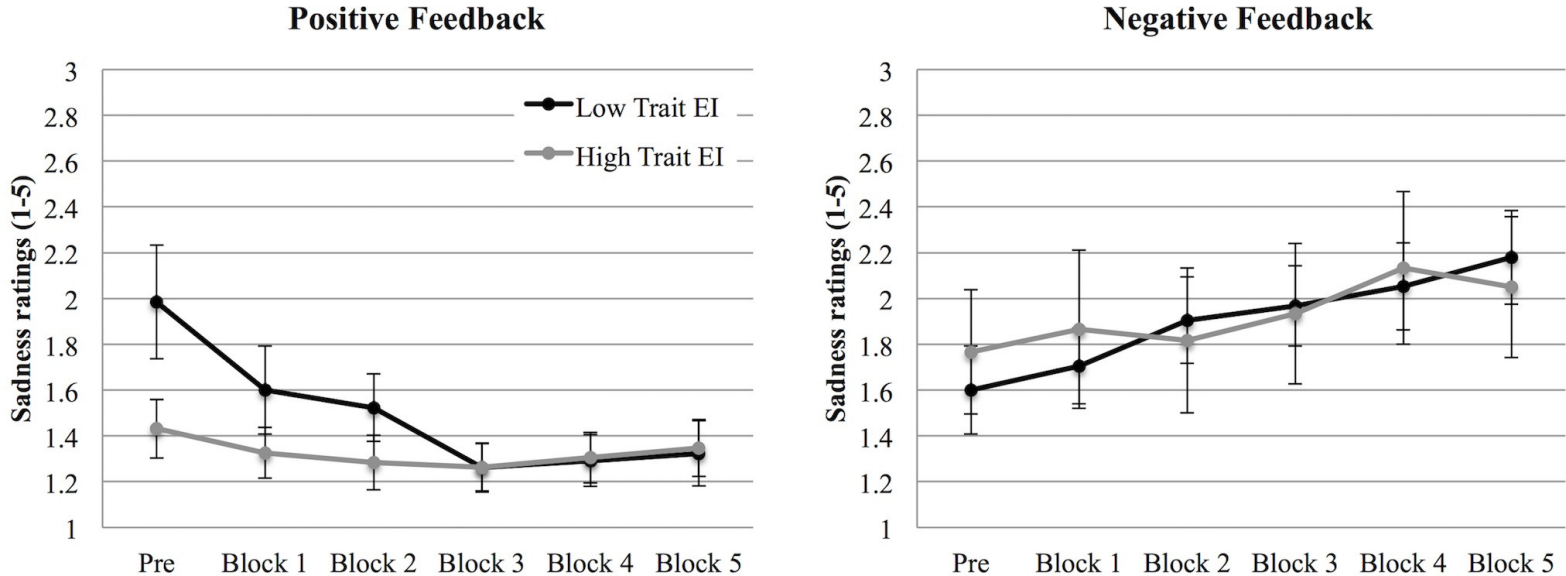
Sadness (Study 1). Sadness intensity at the beginning of the experiment (PRE) and after each block in the negative (left panel) and in the positive (right panel) feedback conditions for low and high trait EI participants. Error bars indicate 1 standard error above or below the mean.

First of all, we found a main effect of Feedback on the sadness felt by participants across blocks, *F* (1, 59) = 7.81, *p* = .007, η^2^_p_ = .117, showing higher overall sadness in the negative feedback condition than in the positive feedback one. Moreover, a Repetition X Feedback interaction emerged, *F* (5, 295) = 14.90, *p* < .001, η^2^_p_ = .202, that showed two different trends in the emotional experience of sadness in the positive and in the negative feedback conditions. Supporting H1, while in the negative condition sadness increased during the task, it decreased in the positive condition (see Fig 3). Finally, a Repetition X Feedback X trait EI level interaction emerged, *F* (5, 295) = 3.85, *p* = .002, η^2^_p_ = .061.

In order to explore this latter interaction, we performed two separate ANOVAs, one for the negative feedback condition and one for the positive feedback condition, respectively. In the negative feedback condition, we found only a Repetition effect, *F* (5, 145) = 6.52, *p* < .001, η^2^_p_ = .184, that again showed an overall increase of sadness intensity across blocks, irrespective of the trait EI level (Fig 3, left panel). In the positive feedback condition, we found both a Repetition effect, *F* (5, 150) = 10.83, *p* < .001, η^2^_p_ = .265, and a Repetition X trait EI level interaction, *F* (5, 150) = 5.41, *p* < .001, η^2^_p_ = .153. Interestingly, the main effect of Repetition was qualified by a significant interaction that, supporting H3, revealed that while the positive feedback did not influence sadness in high trait EI participants (who were characterized by an overall lower sadness), it reduced sadness intensity in low trait EI participants (Fig 3, right panel). In particular, a significant decrease in sadness intensity emerged in low trait EI individuals across blocks (*ps* < .031), especially between the measurement performed before the beginning of the experiment and the first block and between the second and the third block.

The guilt analyses revealed a main effect of Feedback, *F* (1, 59) = 8.50, *p* = .005, η^2^_p_ = .126, that revealed higher guilt scores in the negative feedback condition. The main effect of Feedback was further qualified by a Repetition X Feedback interaction, *F* (5, 295) = 11.62, *p* < .001, η^2^_p_ = .165, indicating that this difference varied across blocks in the two conditions. In line with H1, in the negative condition, guilt increased during the task, whereas it decreased in the positive condition. Finally, a Repetition X Feedback X trait EI level interaction emerged, *F* (5, 295) = 2.24, *p* = .051, η^2^_p_ = .037.

**Fig 4:**
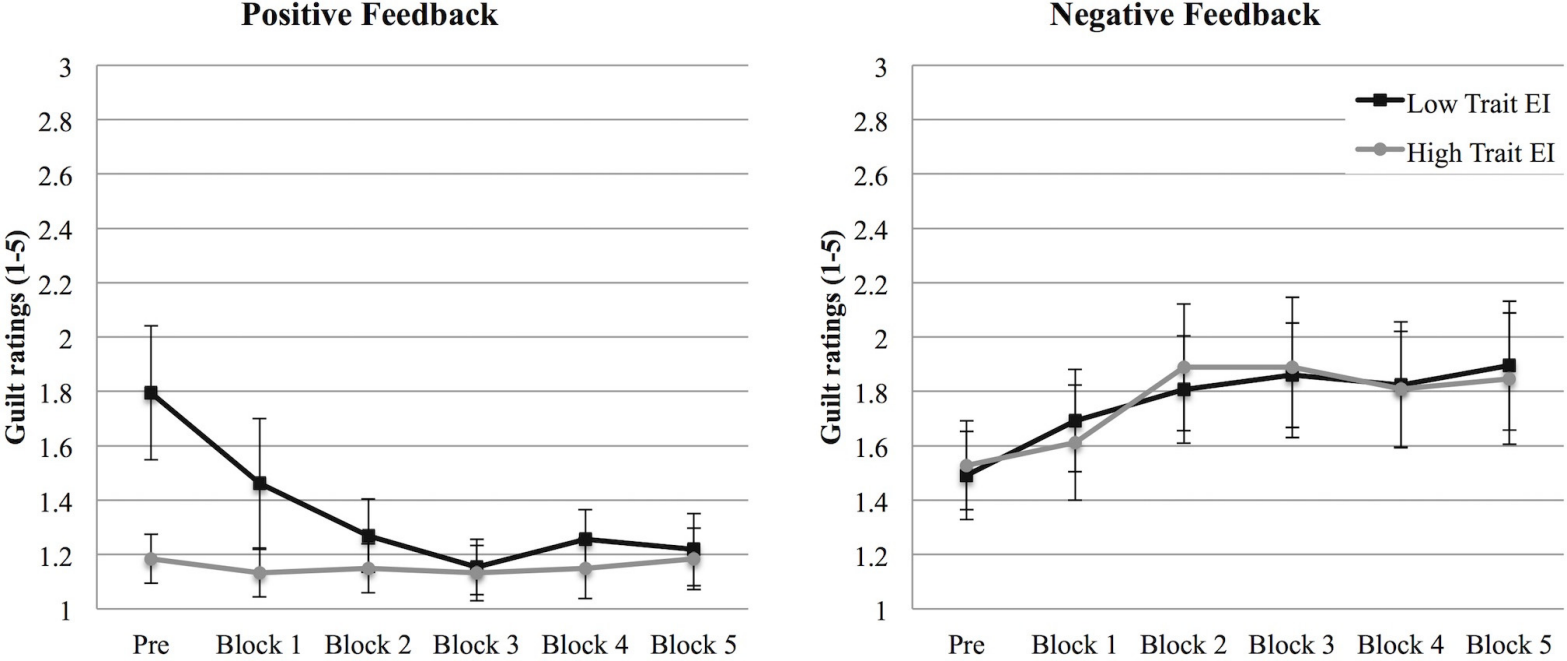
Guilt (Study 1). Guilt intensity at the beginning of the experiment (PRE) and after each block in the negative (left panel) and in the positive (right panel) feedback conditions for low and high trait EI participants. Error bars indicate 1 standard error above or below the mean.

Two separate analyses performed on the two feedback conditions showed that in the negative condition, a Repetition effect emerged, *F* (5, 145) = 4.96, *p* < .001, η^2^_p_ = .146 (Fig 4, left panel). This effect showed that the negative feedback affected the experience of guilt, enhancing its intensity across the five blocks, irrespective of trait EI. In the positive feedback condition, we found both a main effect of Repetition, *F* (5, 150) = 7.96, *p* < .001, η^2^_p_ = .210, and a Repetition X trait EI interaction, *F* (5, 150) = 6.70, *p* < .001, η^2^_p_ = .183. As shown in Fig 4 (right panel), confirming H3, high trait EI participants did not experience a different guilt intensity from the initial measurement across blocks; on the contrary, for low trait EI participants, guilt intensity decreased throughout the task. In particular, guilt intensity was significantly lower at the end of each of the five blocks in comparison with the measurement performed before the beginning of the experiment, *ps* < .013.

A main effect of Feedback, *F* (1, 59) = 6.95, *p* = .011, η^2^_p_ = .105, emerged on joviality ratings, which showed, supporting H1, an overall lower joviality in the negative feedback condition. Moreover, analyses showed a main effect of trait EI level, *F* (1, 59) = 5.55, *p* = .022, η^2^_p_ = .086, that testified to an overall higher joviality in high trait EI participants than in low trait EI participants.

**Fig 5:**
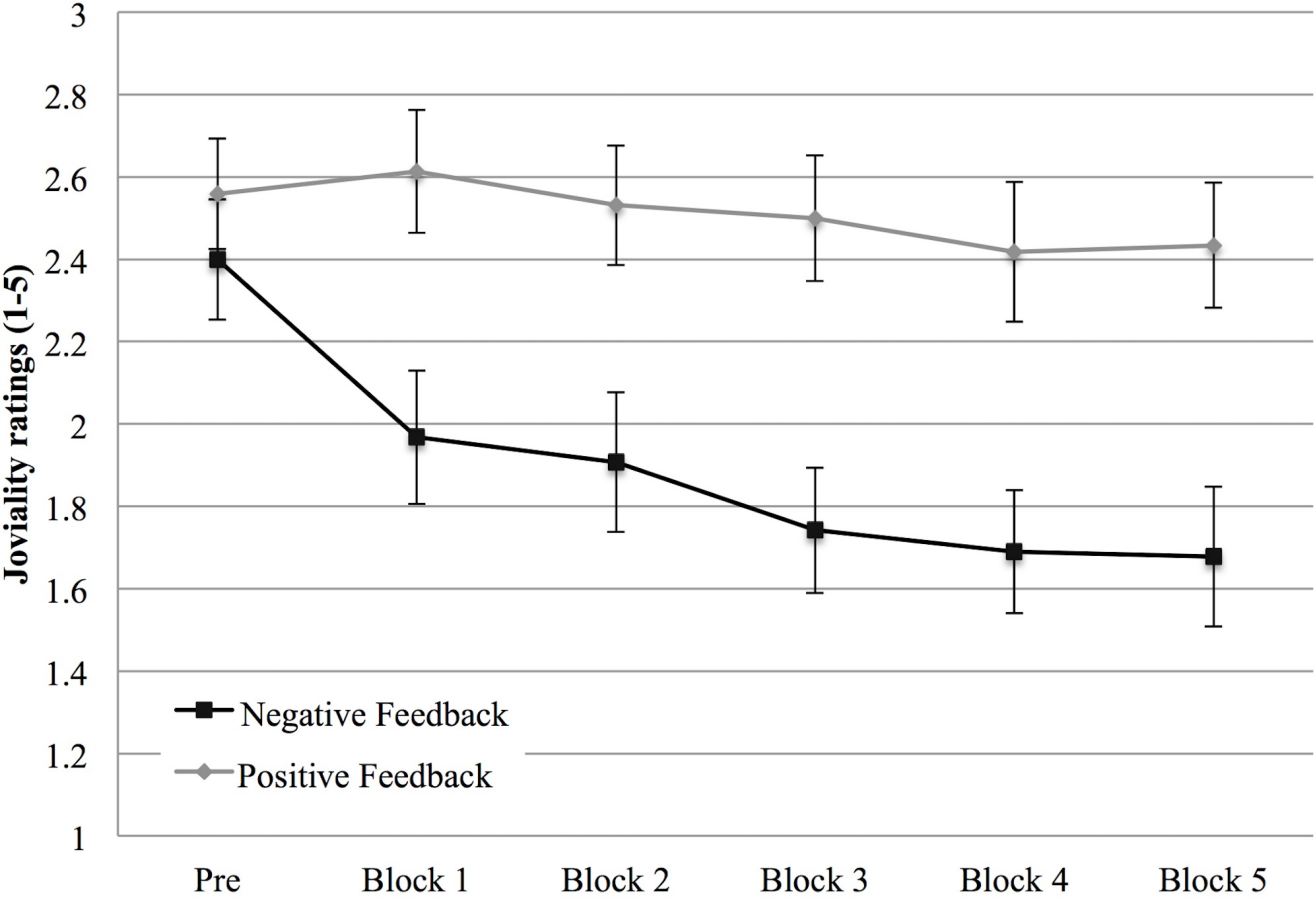
Joviality (Study 1). Joviality intensity at the beginning of the experiment (PRE) and after each block in the positive and negative feedback condition. Error bars indicate 1 standard error above or below the mean.

Finally, we found both a Repetition effect, *F* (5, 295) = 12.94, *p* < .001, η^2^_p_ = .180, and a Repetition X Feedback interaction, *F* (5, 295) = 6.33, *p* < .001, η^2^_p_ = .097. This interaction showed that while joviality did not change from the first measurement in the positive feedback condition, it decreased in the negative feedback condition (Fig 5).

A main effect of Feedback, *F* (1, 59) = 7.36, *p* = .009, η^2^_p_ = .111, emerged on self-assurance ratings, which showed, fully confirming H1, a general lower self-assurance in the negative feedback condition than in the positive feedback condition. Moreover, we found a main effect of trait EI level, *F* (1, 59) = 8.62, *p* = .005, η^2^_p_ = .127, indicating a greater self-assurance in high trait EI individuals than in low trait EI ones.

**Fig 6:**
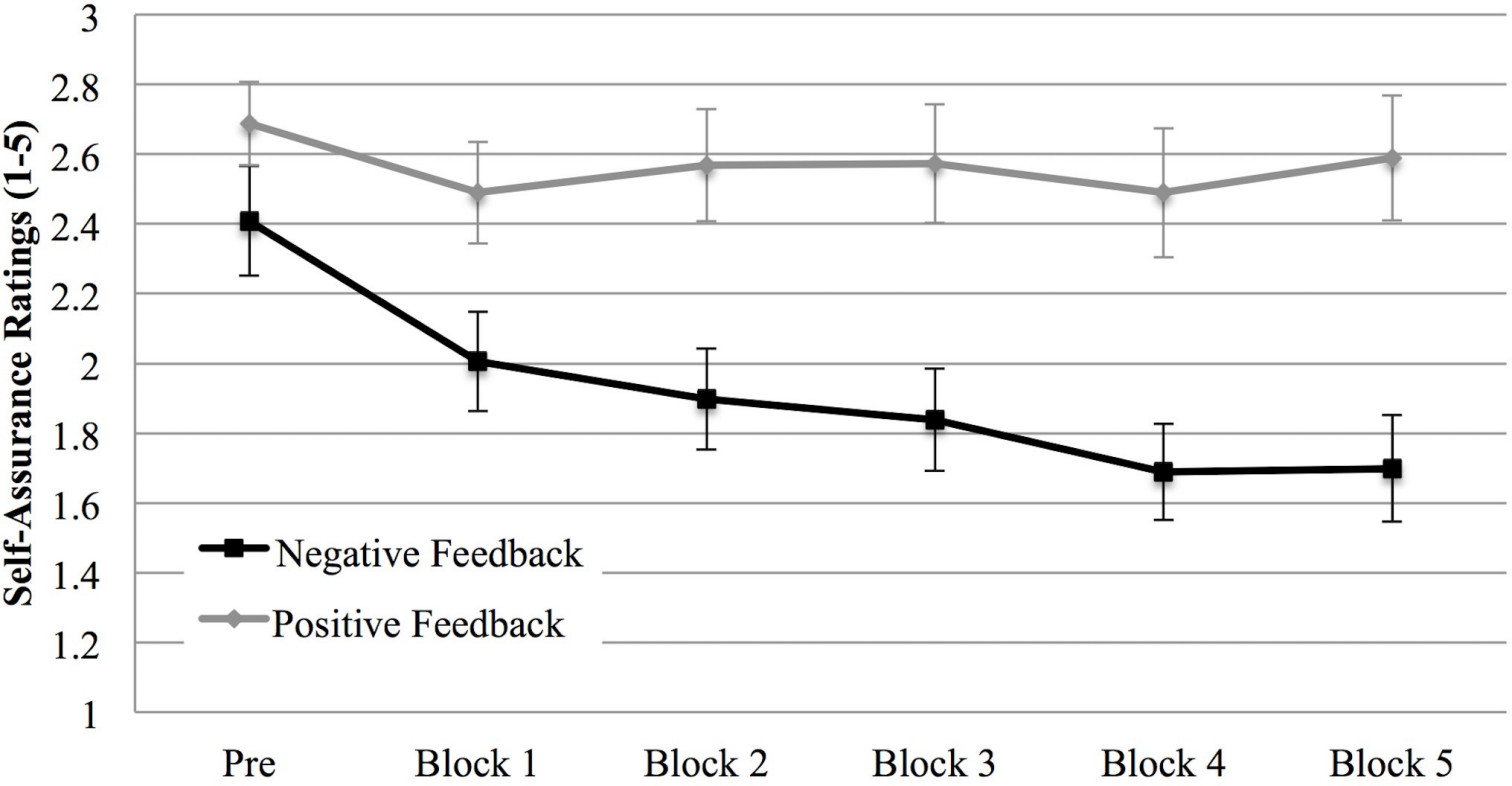
Self assurance (Study 1). Self assurance at the beginning of the experiment (PRE) and after each block in the positive and negative feedback condition. Error bars indicate 1 standard error above or below the mean.

Finally, a main effect of Repetition, *F* (5, 295) = 12.17, *p* < .001, η^2^_p_ = .171, and a Repetition X Feedback interaction, *F* (5, 295) = 5.55, *p* < .001, η^2^_p_ = .086, emerged. This interaction showed that while self-assurance did not change in the positive feedback condition, it changed across blocks in the negative feedback condition (*ps* < .027), in particular from the initial measurement to the first block and from the first block to the second one (Fig 6).

**Fig 7:**
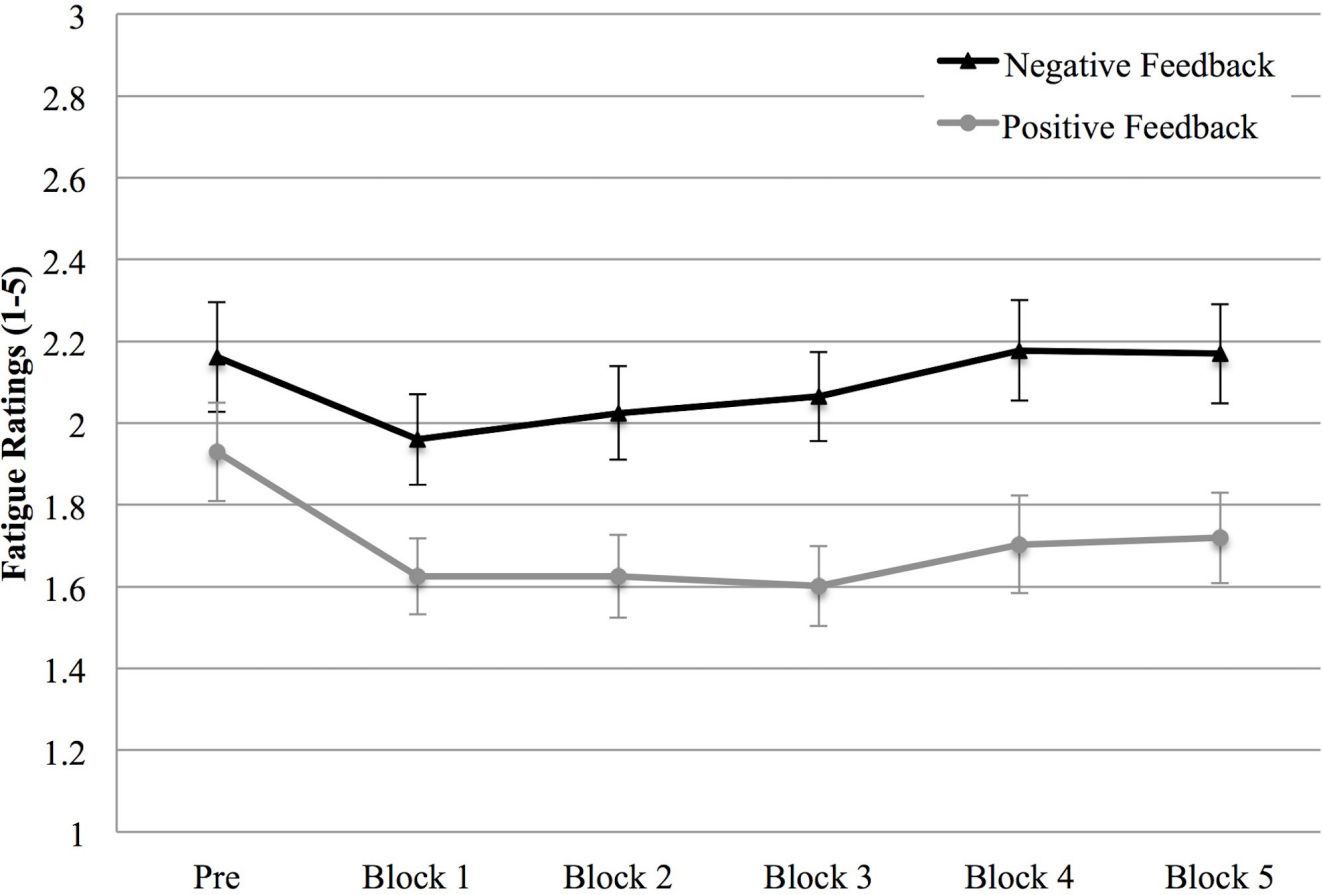
Fatigue (Study 1). Fatigue intensity at the beginning of the experiment (PRE) and after each block in the positive and negative feedback condition. Error bars indicate 1 standard error above or below the mean.

Analysis of the level of fatigue before the computer task and after each block supported H1 and revealed a main effect of Feedback, *F* (1, 59) = 7.36, *p* = .009, η^2^_p_ = .111, showing that participants experienced a higher fatigue in the negative feedback condition than in the positive one (Fig 7). Moreover, a main effect of Repetition emerged, *F* (1, 59) = 7.36, *p* = .009, η^2^_p_ = .111, showing a decrease in fatigue in the first three blocks compared to the measurement carried out before the computer task, *ps* < .003.

#### Power analysis

In order to ensure that our sample size was sufficient to detect an adequate effect, we computed post hoc power analyses using G*Power 3 Software. Results revealed that our total sample size of 63 participants was sufficient to detect with a .99 power a medium effect size (F = .238) [50]. The power analysis was conducted on the hypothesized three-way interaction predicting accuracy.

### Discussion

Confirming our set of hypotheses, we found that the manipulation of the feedback conditions (positive vs. negative) led to different levels of perceived efficacy among participants. In particular, results showed that negative feedback induced a progressive increase of negative affective states. Overall, participants felt higher levels of sadness, guilt, and fatigue, and a decrease in joviality and self-assurance after the repeated presentation of negative feedback. This pattern indicates that the intensity of negative affective states induced by the negative feedback increased block after block. Moreover, we found that this increasing negative state affected participants’ performance, leading to a reduction of responses accuracy. On the basis of these results, we argue that the repeated presentation of negative feedback could induce in participants an increasing sense of inefficacy that could led to negative affective states and to a reduction of the motivation to help (reflected in the accuracy decrease). Future studies should test our assumptions, replicating the present findings in accuracy and demonstrating a reduction in the motivation to help. These results are consistent with previous work on the relation between sense of efficacy and prosocial behavior. Axelrod and Lehman, for example, found that individuals with high levels of perceived efficacy in relation to environmentally-responsible behavior were more inclined to behave in an environmentally responsible manner [51]. Moreover, our results are consistent with the “drop in the bucket” effect [25–26]. According to this effect, people reduce their willingness to help when the victim’s suffering is seen as part of a bigger problem and therefore their efficacy in helping is perceived as lower. Consistently, the repeated presentation of negative feedback induced in participants an increasing sense of helplessness, reducing their motivation to help. However, unlike the "drop in the bucket" effect, where people’s sense of inefficacy is determined by the perception of the uselessness of their behavior with respect to the vastness of the problem, in the present study the sense of inefficacy is restricted to an intra-individual domain, and is determined by the personal failure in helping others.

More important for the purpose of this work, we found that trait EI interacted with the valence of feedback in determining participants’ performance. With regards to the negative condition, we did not find any difference in the intensity of affective reactions among high and low trait EI individuals. These results suggest that the repeated presentation of negative feedback induced in participants a sense of inefficacy, irrespective of trait EI level. The perceived inefficacy was reflected in an increasing intensity of sadness, guilt, and fatigue, and in a decrease of joviality and self-assurance in all participants. However, the results showed that high and low trait EI participants performed differently after the presentation of negative feedback. In particular, after an initial low accuracy, high trait EI participants progressively increased their performance. On the contrary, low trait EI participants progressively decreased their performance across blocks. These results provide support to the modulatory role played by trait EI on the increasing sense of inefficacy triggered by negative feedback. Despite the negativity induced by the increased sense of inefficacy, high trait EI participants were able to better manage their affective reactions than low trait EI participants, modulating the impact of their emotions on the performance and maintaining a higher level of motivation to help (reflected in performance improvement). On the contrary, low trait EI participants were totally at the mercy of their negative feelings (i.e., inefficacy).

Moreover, we found that trait EI also affected participants’ affective states in the positive feedback condition. Even if we did not find differences in performance between high and low trait EI in the positive condition, we found that in low trait EI participants, the repeated presentation of a positive feedback decreased the intensity of their negative affective reactions (sadness and guilt). This result, coupled with an overall more positive affective state in high trait EI participants (testified by higher levels of joviality and self-assurance), could signal a higher and more stable positive mood attitude in these individuals. While positive feedback had an impact on low trait EI individuals’ affective states, no effect was found for high trait EI individuals, likely because of their usually higher optimism and positivity. Indeed, happiness levels of high trait EI people were higher than happiness levels of low trait EI people, and there is also preliminary evidence that high trait EI individuals experience a more positive valence after seeing pictures of neutral faces [45].

However, reasonable doubt could arise from the analysis of these results: do these effects depend exclusively on the altruistic nature of the task or are they products of other variables not directly related to helping behavior or to the presence of a victim in need? In other words, it remains unclear whether the change in the affective states, the differences between high and low trait EI participants, and the difference in the performance stem from the motivation to help the victim or whether these effects are the mere reactions to feedback informing participants’ about their success or failure in the task. To test this alternative explanation, we conducted a second study to explore whether these effects also emerged when the performance in the computer task was not related to helping children in need.

## Study 2: Trait EI and participants’ performance

Study 2 provided a control situation for exploring participants’ behavior when facing failure or success in a situation unrelated to helping others in need. Unlike Study 1, the goal was not to help a child in need by clicking on his/her picture, but only to click accurately and quickly on a black square. Participants completed five blocks with 30 trials in each block. Study 2 adopted the same manipulations as Study 1, with either positive or negative feedback presented at the end of each block informing participants that they had been either unable or able to achieve the goal (accurately clicking on the black squares in less than 500 ms). As in Study 1, we measured participants’ affective reactions at the beginning of the experiment and after each block using the PANAS-X Scale [47], and assessed trait EI at the end of the experiment using the TEIQue-SF [48].

We hypothesized that the lack of a helping situation should not induce changes in participants’ affective states. More specifically, we hypothesized that, contrary to Study 1, the manipulations used in Study 2 should be less affectively-charged, and consequently they should not impact participants’ affective states. As a consequence of these assumptions, neither the positive nor the negative feedback condition should be able to alter (positively or negatively) participants’ sense of efficacy. Therefore, no effect of the feedback condition on participants’ affective states should emerge across the blocks. On the basis of the less intense affective reactions to failure or success, we hypothesized that participants (and in particular, high trait EI participants) should not be motivated to enhance their performance as a reaction to their affective states.

### Method

#### Ethical statement

The procedure was approved by the Ethical Committee for Research in Psychology (Area 17) of the University of Padova (protocol 1249 N. 5D60A256530D615E135C5B9017C34611).

#### Participants

Fifty-nine undergraduate students (53.3% females; mean age 24.52 years, *SD* = 3.76) participated in the study. They were all volunteers and were recruited during class hours. They were then scheduled for individual experimental sessions in the laboratory. Written consent was obtained from each participant upon they arrival in the laboratory as required by the regulation of the Ethical Committee of the University of Padova regarding cognitive/behavioral studies involving adult human participants.

Participants were randomly assigned to one of the two experimental conditions: 30 in the positive feedback condition and 29 in the negative feedback one. They were asked to complete the experimental task, answer a set of questionnaires, and provide demographic information about their gender and age.

#### Questionnaires

Both TEIQue-SF [48] and PANAS-X [47] were used in Study 2. Once again, TEIQue-SF showed a good reliability: α = .89. The five specific affective dimensions measured by the PANAS-X showed a good internal reliability as well, with a Cronbach’s alpha ranging from .69 to .93 for the joviality, guilt, self-assurance, and sadness affect. The reliability for the fatigue dimension was slightly lower, ranging from .53 to .76 across the five experimental blocks (see Table 2 for reliability measures of the PANAS-X scales).

**Table 2:** Reliability (Cronbach’s alpha) of the PANAS-X affect scales administered at the beginning of Study 2 (PRE) and at the end of the five blocks.

PANAS-X Affect	PRE	Block 1	Block 2	Block 3	Block 4	Block 5
Sadness	.724	.780	.742	.824	.740	.836
Guilt	.784	.693	.742	.848	.823	.818
Joviality	.869	.901	.932	.921	.917	.935
Self-assurance	.813	.845	.809	.876	.861	.882
Fatigue	.615	.537	.703	.715	.744	.762

#### Procedure

The experimental design used in the present study was identical to the design used in Study 1. TEIQue-SF and PANAS-X were administered at the same stages as in the earlier study. The same equipment and the same stimuli presentation settings were also used (e.g., stimuli presentation modality and dimension).

Also in Study 2, each block included 30 trials, but the stimulus (a black square) remained the same across all five blocks. Participants were instructed to click with the left button of the mouse on the black square randomly displayed in one of the four corners of the computer screen. They were told they could obtain a hypothetical score only if they were able to accurately click on the black squares with an average response time lower than or equal to 500 ms in each block. The two experimental conditions were manipulated as follows. In the negative feedback condition, participants received the same feedback at the end of each of the five blocks informing them that they did not succeed in clicking accurately on the black square within the time limit (“You did not complete the block successfully”). In the positive feedback condition, the same feedback at the end of each block stated that they succeeded in clicking accurately on the square within the time limit (“You completed the block successfully”). In both conditions, the presentation of the feedback at the end of each block was preceded by a slide on screen for 3000 ms that informed participants that their result was being computed. As in Study 1, the feedback provided to the participants in Study 2 was fake and not related to their actual performance.

### Results

An initial analysis again showed no effect of the feedback condition (positive or negative) on the trait EI level, *t* (57) = 1.04, *p* = .301.

#### Performance

**Fig 8:**
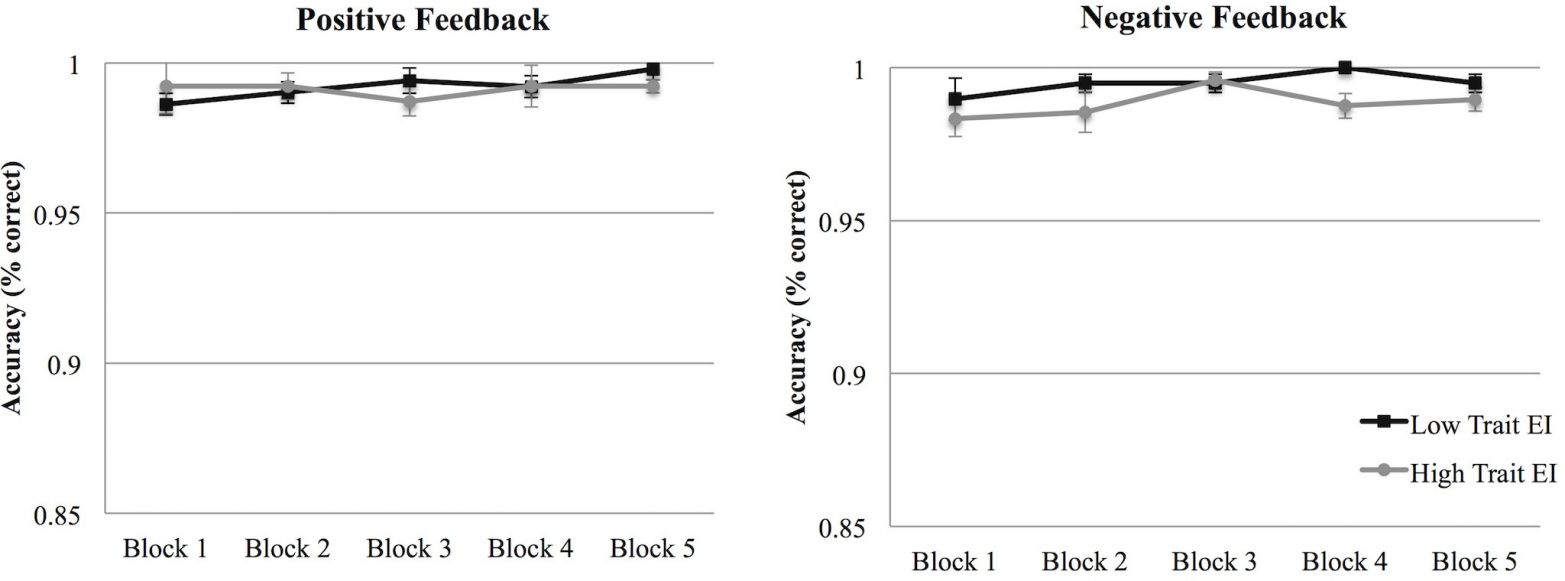
Accuracy (Study 2). Accuracy percentages in the negative (left panel) and in the positive (right panel) feedback conditions across the 5 blocks for low and high trait EI participants. Error bars indicate 1 standard error above or below the mean.

In the analysis performed on response accuracy, no significant effect emerged. The response accuracy did not significantly differ across blocks, between the different feedback conditions, or between the two trait EI levels (Fig 8).

**Fig 9:**
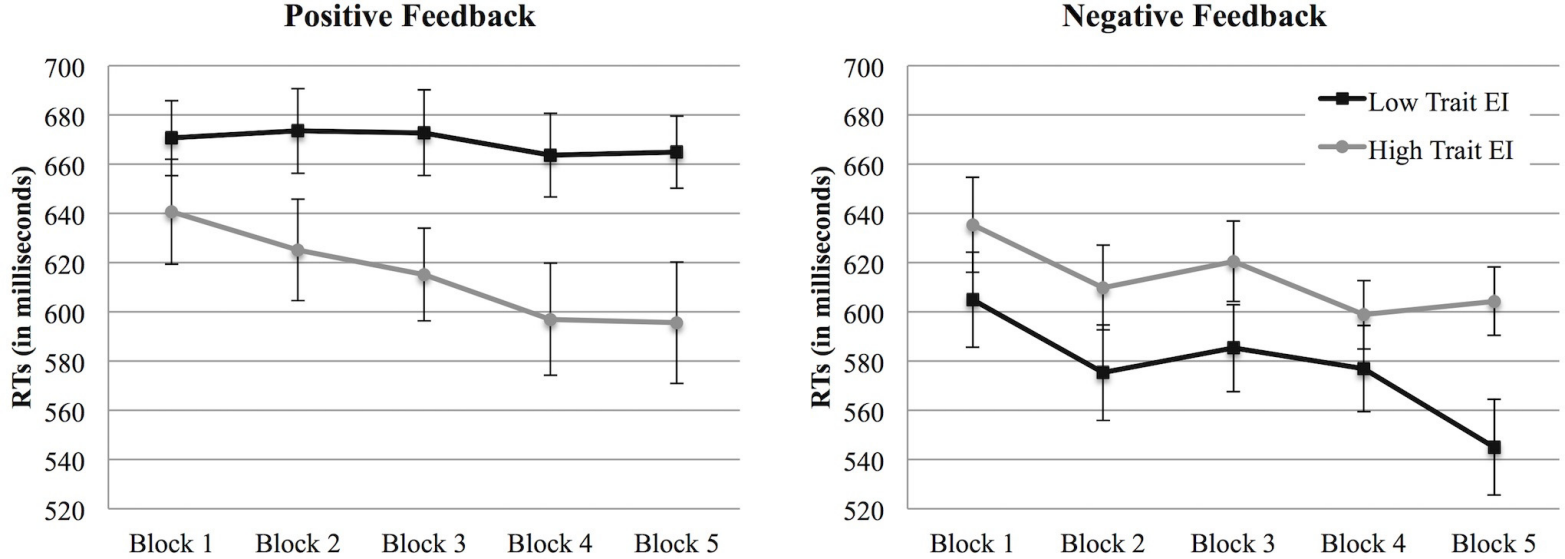
Reaction times (Study 2). Reaction times in the negative (left panel) and in the positive (right panel) feedback conditions across the 5 blocks for low and high trait EI participants. Error bars indicate 1 standard error above or below the mean.

The analysis performed on reaction times showed a Repetition effect, *F* (4, 220) = 9.94, *p* < .001, η^2^_p_ = .153, that highlighted a general reduction in reaction times across blocks. Additionally, an effect of Feedback emerged, *F* (1, 55) = 7.79, *p* = .007, η^2^_p_ = .124, showing overall faster reaction times in the condition with negative feedback, *M* = 597.51, *SD* = 65.33, than in the condition with positive feedback, *M* = 645.52, *SD* = 67.28. The main effect of Feedback was further qualified by a Feedback X trait EI level interaction, *F* (1, 55) = 7.48, *p* = .008, η^2^_p_ = .120. While in the positive feedback condition high trait EI participants responded faster than low trait EI participants (Fig 9, right panel), in the negative feedback condition this trend reversed, with low trait EI participants responding faster across blocks than their trait EI counterparts (Fig 9, left panel). Finally, high trait EI participants did not change their performance trend between the two conditions, showing the same reaction times (and a decrease across scenarios) both in the negative feedback and in the positive feedback conditions; on the contrary, low trait EI participants reduced their reaction times in the negative condition compared to the positive one.

#### Affect

The sadness analyses revealed only a trait EI effect, *F* (1, 54) = 4.18, *p* = .046, η^2^_p_ = .072, that showed an overall higher sadness level in low trait EI participants, *M* = 1.63, *SD* = .55, than in high trait EI ones, *M* = 1.38, *SD* = .37. Neither the effect of repetition nor the effect of feedback condition emerged.

Similarly to the sadness results, analyses of participants’ guilt showed only a trait EI effect, *F* (1, 54) = 5.72, *p* = .020, η^2^_p_ = .096, that showed an overall higher guilt intensity in low trait EI participants, *M* = 1.38, *SD* = .46, than in high trait EI ones, *M* = 1.16, *SD* = .21.

In the joviality analyses, we found a Repetition effect, *F* (5, 270) = 63.93, *p* < .001, η^2^_p_ = .128, that showed a general decrease across blocks in joviality intensity.

An initial result emerged from self-assurance analyses with the main effect of Repetition, *F* (5, 270) = 6.29, *p* < .001, η^2^_p_ = .104, showing an overall decrease of self-assurance across blocks. Moreover, a main effect of trait EI emerged, *F* (1, 54) = 6.29, *p* = .015, η^2^_p_ = .104, indicating an overall lower self-assurance level in low trait EI participants, *M* = 2.48, *SD* = .88, than in high trait EI ones, *M* = 3.00, *SD* = .72.

The analysis of the level of fatigue before the computer task and after each block revealed a main effect of Repetition, *F* (5, 270) = 9.52, *p* < .001, η^2^_p_ = .150. Moreover, a Repetition X trait EI effect emerged, *F* (5, 270) = 3.55, *p* = .004, η^2^_p_ = .062. In particular, while for low trait EI participants fatigue decreased, especially from the first block to the second one, for high trait EI participants it remained low in all experimental blocks.

#### Power analysis

In order to ensure that our sample size was sufficient to detect an adequate effect, we computed post hoc power analyses using G*Power 3 Software. Results revealed that our total sample size of 59 participants was sufficient to detect with a .99 power a good effect size (F = .369) [50]. The power analysis was conducted on the interaction between trait EI and feedback predicting response times.

### Discussion

In Study 2, no effect of emotional intelligence or feedback condition emerged on response accuracy. However, an interaction effect between these two variables emerged in participants’ reaction times. The results showed that high trait EI participants responded faster than their low trait EI counterparts in the positive feedback condition, while low trait EI participants were faster during the failure condition.

Even if we again found that the nature of the feedback affected participants’ performance, in this second study this effect was evident only in low trait EI participants. While reaction times among high EI did not vary across the two conditions, showing an overall decrease both in the negative and in the positive feedback conditions, low trait EI participants showed a decrease in reaction times only in the negative feedback condition. Unlike Study 1, in Study 2 we cannot infer that the feedback affected the individual sense of efficacy. No effect of feedback emerged on participants’ affective reactions. Consistent with the result of Study 1, the affect analyses showed an overall effect of trait EI on participants’ affective states, with high trait EI participants generally characterized by lower negative affect (sadness, guilt, and fatigue—the last in interaction with the blocks’ repetition) and higher self-assurance levels than low trait EI participants. Even if the experimental situation was characterized by an overall decrease of joviality and self-assurance across blocks, these effects testify that the manipulations used in Study 2 were affectively poor. That is, in this second study, neither the positive nor the negative feedback impacted participants’ emotionality, even when they showed high trait EI levels. Since no difference in the affective reactions in the two conditions emerged, the difference in the performance in the two conditions was not dependent on a change in the participants’ affective state. Notwithstanding the lack of differences in affective reactions between the two conditions, we cannot exclude that constantly receiving negative feedback could have induced a feeling of frustration in the participants. Future studies are needed to further explore this emotional state and whether it could affect participants’ performance.

In Study 2, the lack of difference in affectivity between the two conditions did not impact the behavior of high trait EI participants, who are usually characterized by an higher sensitivity to affective cues [29]; they simply did not have any motive (or motivational drive) to change their performance across the two conditions. We can assume that high trait EI individuals mainly react to changes in their affective states or to environmental affective cues. On the contrary, low trait EI participants react to simple external positive and negative feedback that inform them that they should maintain (success) or increase (failure) their performance. If high trait EI participants could be defined as internally-driven individuals, low trait EI participants could be defined as externally-driven individuals. However, the introduction of a more affectively-loaded condition (as in the helping situation explored in Study 1) can drastically change the performance among high and low trait EI individuals, given that affective reactions represent powerful cues that inform us about the contextual situation, and that must be managed in order to react in the most suitable way.

## General Discussion

The comparison between the results of Study 1 and Study 2 gives us a clear picture on the relationship between trait EI and the motivation to help. In particular, the different pattern of results emerged in the two studies shows that the effect of trait EI on performance, as it emerged in Study 1, was properly related to the helping situation and not to a general reaction to success and failure. The different impact of positive and negative feedback in the two studies showed that a helping situation can be considered a highly affectively-charged context, where individuals have to deal with the affective reactions stemming from their perceived effectiveness or ineffectiveness in helping suffering others. The two feedback-based manipulations adopted in the two studies were perceived very differently. In Study 1, the feedback led to intense affective reactions, whereas in Study 2, it did not induce equally intense affective reactions. While in a helping situation a high level of trait EI emerged as a central factor in producing a concrete helping act, in Study 2, a low level of trait EI was responsible for changing individuals’ performance. When a situation lacks affective information, high trait EI individuals seem to have no information to react to. However, the results of Study 1 support the conclusion that the effectiveness in helping a victim in need induces emotions intense enough to become salient information that high trait EI people can use to regulate their behavior.

The results of Study 1 showed that the individual differences in trait EI is an important determinant of the motivation to help suffering others, particularly after the presentation of a negative feedback. We found that after an initial lower performance in accuracy, high trait EI participants progressively increased their performance, while low EI participants progressively decreased their performance across blocks. The initial lower performance of high trait EI participants in Study 1 could be explained on the basis of past results showing a greater sensitivity to environmental affective cues in these individuals. Petrides and Furnham, for example, showed that high trait EI individuals are more sensitive to the effects of mood induction than their lower scoring counterparts [29]. It is reasonable to believe that the negative feedback could have induced an initial stronger effect on more sensitive individuals (i.e., high trait EI participants), who have then been able to react more efficaciously to the negativity induced by this condition. This more effective response to negativity may stem from a better management of affective reactions in high EI people. Emotional management and, in particular, stress management is central to any model of EI. In particular, trait EI is considered a protective factor against stress and is associated with higher optimism [52]. Despite an increase of negativity experienced by all participants in the negative feedback group in Study 1, the superior emotion management skills in high trait EI people emerged in the ability to reduce the impact of the increasing negativity on their performance. Emotion management, indeed, determines how a person’s emotions are dealt with once activated [53]. Our results confirmed both a more efficacious response to stressors (negative feedback) and an overall more positive affective attitude in high trait EI participants. Therefore, Study 1 showed that even if high trait EI people are more sensitive to environmental emotional cues, they seem more able to manage the impact of their affective reactions on their behavior, at the same time preserving their naturally positive disposition. On the contrary, the emotionally-poor situation proposed in Study 2 did not require emotion management by high trait EI participants since no change in individual affective reactions emerged.

Based on these results, we suggest that emotion management is as important as affective reactions in determining motivations for helping suffering others. Indeed, these results showed that specific affective reactions do not always coincide with specific helping behaviors (as revealed by the different performances in low and high trait EI participants in Study 1), but the individual emotional disposition (trait EI) and its management play important roles. Our data are in agreement with Dickert et al. [16], who highlighted the role of affect management in the motivation to help. Moreover, adopting a dual information-processing framework, we could assume that high trait EI individuals are characterized by a more efficacious integration and interaction between System 1 and System 2. While the behavior of low trait EI people should be primarily driven by System 1, high trait EI participants appear able to use (albeit only in an implicit way) a more deliberative assessment of the victims and the situation (System 2), reducing the impact of the immediate affective reactions.

Moreover, Study 1 represents the first work that tested the effect of feedback on repeated charitable efforts. This paradigm could be useful in exploring the factors influencing charitable campaigns that require long-lasting helping behaviors. Our results, for example, could in part explain the reduction of some prosocial actions as a consequence of their repeated failure; an example of this effect could be the inefficacy sometimes perceived in helping drug addicts who often tend to relapse in drug problems. We call this the “Sisyphus effect;” like the mythological figure condemned to repeat forever the same meaningless task of pushing a boulder up a mountain, only to see it roll down again, our participants felt an increasing sense of inefficacy and a repeated failure of their actions in reaching the desired result, notwithstanding their efforts. However, unlike Sisyphus, who was condemned to repeat his action for the rest of eternity, our participants could implicitly reduce their efforts, as evidenced by the measured performance. Unlike the classical methods used to test the motivation to help (these usually ask participants directly about their willingness to help or donate [16]), the task devised for the present work tested helping behavior implicitly, measuring people’s efforts through accuracy and reaction times. We believe that this method could represent a useful alternative to more traditional measures of willingness to help, in that it is not affected by social rules, like social desirability, and it can measure such behavior in a more sensitive way using two different objective measures: accuracy and reaction times. However, the feedback provided in both Study 1 and Study 2 was in fact a fake score (i.e., unrelated to participants’ actual performance) and was used to induce a sense of efficacy/inefficacy. Assessing the extent to which participants believed the cover story, as well as the extent to which they felt their donation can indeed benefit others in need may provide further insights on the relationship between feedback and motivation to help. In particular, a manipulation check could help understanding the level of involvement of the participants in the task and lend further support to the effectiveness of the manipulation. Moreover, given the relatively complex results emerged from the interaction effects found in the present studies, we call for future studies replicating the findings showed by the present paper.

A final consideration must be given to the differences in accuracy and reaction times results that emerged from the two studies. Differently from Study 2, in Study 1 the main effects on participants’ performance emerged in the accuracy scores. Moreover, while in Study 1 only an overall decrease of the response times across blocks was found, Study 2 showed a difference between high and low trait EI participants. We suggest that these differences in the results might depend on the different affective load required by the two conditions (positive and negative feedback) in Study 1 and Study 2. While the helping situation used in Study 1 induced a high affective load for the participants, the one presented in Study 2 was relatively simple to elaborate. The high accuracy rates and the response speed emerged in Study 2 highlighted that the task completed by participants (accurately clicking on the stimuli with an average response time lower than or equal to 500 ms in each block) was relatively simple. As a consequence, the affective load hypothesis is supported by the difference in the accuracy rates and reaction times between the two studies. As clearly evident through a comparison of Fig 1 and Fig 8, Study 2 was characterized by higher accuracy rates than Study 1. Moreover, a comparison between Fig 2 and Fig 9 shows that the faster responses in the first study are comparable to the slower responses of the second one (and a statistical analysis confirmed that the reaction times of Study 1 were significantly slower than those of Study 2, *t* (120) = 5.56, *p* < .001). We therefore conclude that in our paradigm, reaction times and accuracy rates are likely to be influenced by the affective load required to process the situation. In particular, in Study 1, the affective load required to manage the helping situation seems to have led to slower reaction times and to an increase in the errors made by participants in clicking on the pictures. While a direct comparison between Study 1 and Study 2 should be done with caution because of the between-subjects nature of the design, the contrast emerging from the results of the two studies highlights the role of the affective reactions elicited by the helping situation used in Study 1 on participants’ performance.

In conclusion, the present paper represents the first work that tested individual differences in managing emotions in helping behavior, adopting a theoretical framework that could account for individual emotional differences in a comprehensive way [42]. We think that trait EI could represent a useful construct for explaining the variability that emerges among people in their motivation to help suffering others. Moreover, we hope that future studies conducted on donation decisions will further test the role played by trait EI in this domain, both measuring people’s motivation to donate and the amount of money they are willing to give in support of a charitable cause.

## Supporting Information

S1 File Instructions for participants and schema of the experimental procedure. (DOCX)

S2 File Informed consent form (English version). (DOCX)

S3 File Modulo di consenso informato (Informed consent form Italian version). (DOCX)

S4 File Briefing and debriefing information. (DOCX)

S5 File Ethics approval document. (DOCX)

S1 Dataset Raw data in csv format for Study 1. (CSV)

S2 Dataset Raw data in csv format for Study 2. (CSV)
