# Supplementary material for: “Give, but Give until It Hurts”: The Modulatory Role of Trait Emotional Intelligence on the Motivation to Help
Source: PLoS One. 2015 Jun 29;10(6):e0130704. doi: 10.1371/journal.pone.0130704 (PMC4487050; doi:10.1371/journal.pone.0130704)
Supplement: S1 File — (DOCX) [file pone.0130704.s001.docx]

**S1. Experimental procedure.**

Instructions for participants and schema of the experimental procedure

During the experimental task you will be asked be to move the mouse pointer on the screen and click on the exact point where you will see appearing the picture of a child. Based on your speed and accuracy you will have the opportunity to gain an amount of money by means of which you could save the life of 5 children. In particular, you will be able to gain money to help each child only if you will respond with accuracy and in less than 500 ms. You will be informed on your performance after each block of trials.
