# Supplementary material for: “Give, but Give until It Hurts”: The Modulatory Role of Trait Emotional Intelligence on the Motivation to Help
Source: PLoS One. 2015 Jun 29;10(6):e0130704. doi: 10.1371/journal.pone.0130704 (PMC4487050; doi:10.1371/journal.pone.0130704)
Supplement: S2 File — (DOCX) [file pone.0130704.s002.docx]

**S2. Informed consent form (English version)**

I declare my consent to volunteer as a participant in the experiment entitled: Emotion regulation and charitable donations.

The goal of the study is the following:

Develop a computer task in which the motivation to help needy individuals is measured through the accuracy and reaction times of the responses. In other words, this research has the goal of going beyond the simple measure of the donation amount that people are willing to give, a problematic measure especially if measured in hypothetical scenarios.

In addition, a second goal of the study is to assess how different levels of emotion regulation influence people’s behavior in situations in which they receive positive versus negative feedback about their performance in the helping task. The task foresees a series of blocks and lasts for about 30 minutes for a total of 5 blocks of 5 minutes each. After every block it is possible to take a short break and rest and it is your decision when to start with the next block.

At the end of every block we will ask you to answer a series of questions about your feelings. Finally, at the end of all five blocks we will ask you to complete a scale for the measurement of trait emotional intelligence.

All information collected during the experiment which can lead to your identification will be kept strictly confidential. The study is anonymous and your responses cannot be linked in any way with your identity, not even by the experimenters who are collecting the data. Below we ask you to sign this informed consent, however this form will be archived in a different location than all the experimental data. In addition, since your responses cannot be linked to your identity, there is no way they can be paired with the present informed consent form.

If, at any moment, you need additional information or clarification about this study, you can contact Doctor Enrico Rubaltelli at the Dipartimento di Psicologia dello Sviluppo e della Socializzazione at the Università di Padova in via Venezia, 8 – 35131 Padova; phone: 049 8276541; email: [enrico.rubaltelli@unipd.it](mailto:enrico.rubaltelli@unipd.it)

Signing this informed consent form, I declare that I have been informed before taking part in the study about my right of leaving the study at any time, without providing any specific reason, without being penalized for that, and with the guarantee that my data will not be used. In addition, I declare that I have been informed about the goal of the study and that my data will be kept anonymous and are protected in compliance with the Law for the protection of personal data (Digs. n. 196/2003). I have been informed that only the researchers running the experiment can get access to my data and only for the seek of the statistical analyses and the anonymous publication of scientific results.

I authorize the researchers responsible for this study to use my responses.

Date:

Signature:
