# Supplementary material for: “Give, but Give until It Hurts”: The Modulatory Role of Trait Emotional Intelligence on the Motivation to Help
Source: PLoS One. 2015 Jun 29;10(6):e0130704. doi: 10.1371/journal.pone.0130704 (PMC4487050; doi:10.1371/journal.pone.0130704)
Supplement: S3 File — (DOCX) [file pone.0130704.s003.docx]

**S3. Modulo di consenso informato (Informed consent form Italian version)**

Con la presente dichiaro di aver acconsentito volontariamente di partecipare allo studio intitolato: Regolazione delle emozioni e donazioni in beneficenza

Lo scopo della studio è quello di:

Sviluppare un compito al computer in cui la motivazione ad aiutare persone bisognose viene studiata misurando tempi di reazione e accuratezza delle risposte. In questo senso, la ricerca si pone l’obiettivo di andare oltre la semplice misura dell’ammontare di denaro che le persone sono disposte a donare, una misura spesso problematica, specialmente quando la richiesta è solamente ipotetica.

Inoltre, uno degli scopi dell’esperimento è quello di vedere come persone con diversa capacità di regolare le emozioni si comportano in situazioni in cui ricevono feedback positivi o negativi riguardo alla propria performance nel compito. Questo compito si compone di diversi blocchi di prove e durerà all’incirca 20 minuti per un totale di 5 blocchi da 4 minuti l’uno. Dopo ogni blocco avrai la possibilità di riposare e deciderai tu quando iniziare un nuovo blocco.

Alla fine di ogni blocco ti chiederemo di rispondere ad una serie di domande relative al tuo umore. Infine, al termine dell’esperimento ti chiederemo di completare una scala che misura l’intelligenza emotiva di tratto.

Ogni informazione ottenuta durante l’esperimento e che possa essere ricondotta alla tua persona rimarrà strettamente confidenziale. Lo studio è completamente anonimo e le tue risposte non possono essere collegate in alcun modo alla tua identità, nemmeno dai ricercatori impegnati in questa ricerca. In basso ti chiediamo di apporre la tua firma ma il modulo di consenso informato sarà custodito in un luogo differente dai questionari cartacei a cui dovrai rispondere. Inoltre, dal momento che le tue risposte non possono essere ricondotte alla tua identità, non c’è alcun modo di collegarle con il presente modulo di consenso informato.

Se, in qualunque momento, hai bisogno di ulteriori informazioni o chiarimenti puoi rivolgerti al dottor Enrico Rubaltelli presso il Dipartimento di Psicologia dello Sviluppo e della Socializzazione dell’Università di Padova in via Venezia 8 – 35131 Padova; telefono: 0498276541; email: [enrico.rubaltelli@unipd.it](mailto:enrico.rubaltelli@unipd.it)

Firmando questo modulo di consenso informato, dichiaro di essere stato informato/a, prima di partecipare al suddetto studio, del mio diritto di interrompere la mia partecipazione allo studio in qualsiasi momento, senza fornire alcuna motivazione, senza alcuna penalizzazione e ottenendo il non utilizzo dei miei dati. Inoltre, dichiaro di essere stato informato dello scopo del suddetto studio e del fatto che i miei dati rimarranno anonimi e protetti secondo il Codice in materia di protezione dei dati personali (Dlgs. n. 196/2003). Sono stato informato che solo le persone che conducono la ricerca potranno avere accesso ai miei dati limitatamente ai fini della loro elaborazione e alla pubblicazione anonima dei risultati a fine scientifico.

Autorizzo i responsabili del presente studio di utilizzare i miei dati.

Data:

Firma:
