# Supplementary material for: “Give, but Give until It Hurts”: The Modulatory Role of Trait Emotional Intelligence on the Motivation to Help
Source: PLoS One. 2015 Jun 29;10(6):e0130704. doi: 10.1371/journal.pone.0130704 (PMC4487050; doi:10.1371/journal.pone.0130704)
Supplement: S4 File — (DOCX) [file pone.0130704.s004.docx]

**S4. Briefing and debriefing information**

Briefing information

Dear Participant, in the next 30 minutes you will be participating in a decision making task in which, based on you performance, you will have the opportunity to accumulate a certain amount of money to help a child in need. The experiment is completely anonymous, and you will not be asked to report at any moment your personal details (i.e. name, email address). Finally, your data will be analyzed only at the aggregate level. If you were to experience any discomfort, or you would like to quit before the end of the experiment, you can do it at any moment without consequences. You can simply notify the experimenter and you will be free to go.

Do you have any questions about the experiment, the way your personal data will be archived or other details relevant to the experimental session?

Debriefing information

Thank you for participating in our experiment. The purpose of the study is to assess whether and to which extent people are motivated to engage in helping behavior over time (here: accurately clicking within a certain time window on the picture of the child displayed on the screen) when provided a positive vs. a negative feedback about their performance. There were two conditions, in the first one, the feedback was always positive (you saved the child), whereas in the second the feedback was always negative (you did not save the child). This feedback was manipulated by the experimenters and had no actual relation with your real performance in the task, therefore you should not judge yourself or your results on the basis of the information received during the experiment. Specifically, irrespective of the actual performance (how quick and accurate the responses are) half of participants was told that they always saved the child, whereas the other half was told that they were not able to save the child. Do you have any questions?  Please, notify us now if you felt any discomfort or any other negative emotions throughout the experiment.

If not, you are free to go.

Thank you.
