# Supplementary material for: “Give, but Give until It Hurts”: The Modulatory Role of Trait Emotional Intelligence on the Motivation to Help
Source: PLoS One. 2015 Jun 29;10(6):e0130704. doi: 10.1371/journal.pone.0130704 (PMC4487050; doi:10.1371/journal.pone.0130704)
Supplement: S5 File — (DOCX) [file pone.0130704.s005.docx]

**S5. Ethics approval document**
